# Disposable screen printed sensor for the electrochemical detection of delta-9-tetrahydrocannabinol in undiluted saliva

**DOI:** 10.1186/s13065-016-0148-1

**Published:** 2016-01-21

**Authors:** Ceri Wanklyn, Dan Burton, Emma Enston, Carrie-Ann Bartlett, Sarah Taylor, Aleksandra Raniczkowska, Murdo Black, Lindy Murphy

**Affiliations:** Oxtox Limited, Warren House, Mowbray Street, Stockport, SK1 3EJ UK

**Keywords:** Delta-9-tetrahydrocannabinol, Δ^9^-THC, Saliva, Mediator, Screen printed electrode, Galvanostatic oxidation, Chronoamperometry, Detection

## Abstract

**Background:**

Cannabis has an adverse effect on the ability to drive safely, therefore a rapid disposable test for Δ^9^-tetrahydrocannabinol (Δ^9^-THC), the psychoactive component of cannabis, is highly desirable for roadside testing.

**Results:**

A screen printed carbon electrode is used for the N-(4-amino-3-methoxyphenyl)-methanesulfonamide mediated detection of Δ^9^-THC in saliva. Mediator placed in an overlayer was galvanostatically oxidized and reacted with Δ^9^-THC to give an electrochemically active adduct which could be detected by chronoamperometric reduction. Detection of 25-50 ng/mL Δ^9^-THC spiked into undiluted saliva was achieved with a response time of 30 s. A trial of the sensors with four cannabis smokers showed sensitivity of 28 %, specificity of 99 % and accuracy of 52 %.

**Conclusions:**

Rapid electrochemical detection of Δ^9^-THC in undiluted saliva has been demonstrated using a disposable sensor, however the sensitivity is lower than acceptable. Further optimization of the assay and sensor format is required to improve the sensitivity of response to Δ^9^-THC.

## Background

In the United Kingdom a 2010 report commissioned by the Department of Transport stated that most drug driving in the UK goes undetected [1]. Two thirds of US trauma centre admissions are due to motor vehicle accidents with almost 60 % of such patients testing positive for drugs or alcohol [2]. Cannabis, cocaine and methamphetamine are the drugs most frequently detected in drivers randomly stopped for roadside drug screening [3–6]. These drugs are frequently abused as recreational drugs due to their stimulant and euphoric effects. Cannabis causes euphoria, somnolence, a change of visual and auditory perception and a decrease in psychomotor abilities. The danger is markedly increased when cannabis is combined with alcohol, which seems to be the case quite frequently. Driving a vehicle while under the influence of cannabis is thus clearly undesirable.

Onsite testing for cannabis and in particular its primary active ingredient Δ^9^-tetrahydrocannabinol (Δ^9^-THC) is routinely performed in urine. Urine testing is not practicable for the roadside screening of a potential drug driver for detecting recent drug use. Oral fluid which contains saliva and other liquid substances present in the oral cavity are of great interest for roadside drug screening. The roadside tests using oral fluid are mainly lateral flow immunoassay systems. Although oral fluid is easy to collect there is considerable inter-sample variability in the fluid matrix that provides issues when developing a testing methodology. The pan-European research project called DRUID (Driving under the Influence of Drugs, Alcohol and Medicines) have called for better screens for cannabis [7]. Testing Δ^9^-THC using four on-site oral fluid drug testing devices gave clinical sensitivities varying between 23 and 81 % [8].

Roadside testing for drugs of abuse has a number of requirements: it needs to be fast, ideally 15–30 s, the same speed as a breath alcohol test, very sensitive, ideally <10 ng/ml (31 nM), it should be non-invasive, with built in controls, difficult to tamper with and be portable. A further important criteria is that the test must be easy to perform by non-laboratory personnel. Some lateral flow devices can give false positive results if they are not kept horizontal during the test procedure. Currently available drug screening products require a minimum of 5–9 min for a test. Test time and cost are currently restricting the roadside drug screening market to <10 % the volume of the alcohol screening market.

The global drug of abuse testing market was valued at $2.9B in 2014. This market is expected to grow at a CAGR of 5.3 % during 2015–2020, to reach $3,9B by 2020, with North America taking the largest market share. The onsite testing market is double the size of the laboratory testing market. In Europe onsite testing had a market size of $346 M in 2014 [9].

Screen printed electrodes are in common use for quick, cheap, disposable tests for a variety of analytes, in particular for measuring blood glucose. Screen printed electrodes are commercially available from a number of suppliers e.g. Dropsens (Spain), Gwent Electronic Materials (UK) and Conductive Technologies Inc. (USA). The application of screen printed electrodes to the detection of drugs of abuse in saliva is therefore of great interest.

There has been few reports of the electrochemical sensing of Δ^9^-THC. The direct electrochemistry of Δ^9^-THC has been reported by absorptive striping voltammetry at a carbon paste electrode [10], and by square wave voltammetry at a glassy carbon electrode [11] and at a paraffin-impregnated graphite electrode [12]. In all cases, pre-concentration of Δ^9^-THC onto the electrode was required to maximize sensitivity. The indirect detection of Δ^9^-THC has been reported using substituted phenols as an electrochemical adaption of the Gibbs reaction [13, 14]. The authors are not aware of any reports of electrochemical detection of Δ^9^-THC in real saliva.

This paper reports a mediated screen printed carbon electrode for the detection of Δ^9^-THC in undiluted saliva using N-(4-amino-3-methoxyphenyl)-methanesulfonamide mediator. The sensor is optimized for response to Δ^9^-THC in undiluted saliva.

## Results and discussion

### Reaction of mediator with Δ^9^-THC

**Fig. 1:**
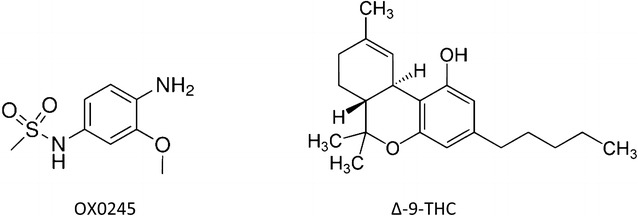
Structures of the mediator OX0245 and Δ^9^-THC

**Fig. 2:**
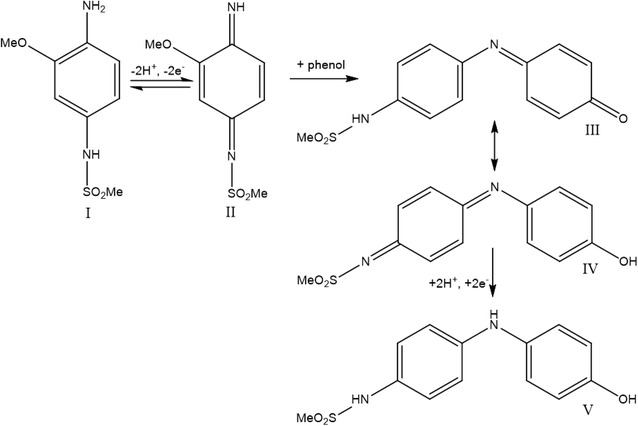
Mechanism for the reaction between OX0245 and phenol

The structures of N-(4-amino-3-methoxyphenyl)-methanesulfonamide (OX0245) and Δ^9^-THC are shown in Fig. 1. The reaction mechanism is shown in Fig. 2 for the reaction between OX0245 and a phenol [15]. Electrochemical oxidation of OX0245 results in oxidation to the diimine, which then reacts with Δ^9^-THC at the 4-position on the phenolic ring, forming an adduct which has two resonance structures, III and IV. The adduct itself can be electrochemically reduced via the diimine of resonance structure IV, and therefore the response to Δ^9^-THC is observed as an increase in reduction current at the diimine reduction potential, since THC, and therefore also the adduct, are relatively insoluble and readily adsorb onto the electrode, giving an enhanced reduction current in addition to the reduction current arising unreacted mediator II which has diffused to the electrode surface.

A single reduction peak is obtained, since the reduction potentials for the diimine of the parent mediator and the adduct are similar. It is unlikely that the quinone form of the adduct III will undergo reduction since it is reported that the quinone form of THC does not undergo electrochemical reduction [12].

**Fig. 3:**
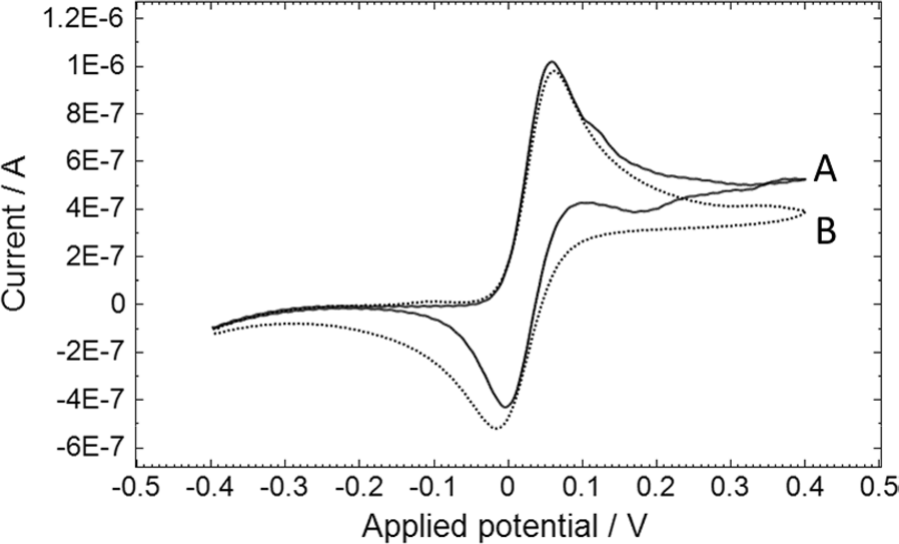
Cyclic voltammetry of OX0245 in the absence or presence of Δ^9^-THC. 15 uL of solution containing 100 ug/mL OX0245 in 0.4 M AMPSO (pH 9.5), 1 M NaCl, 10 % methanol and (*A*) 0 or (*B*) 100 ug/mL Δ^9^-THC was pipetted onto the sensor. The start potential was −0.4 V and the scan rate was 50 mV/sec

**Fig. 4:**
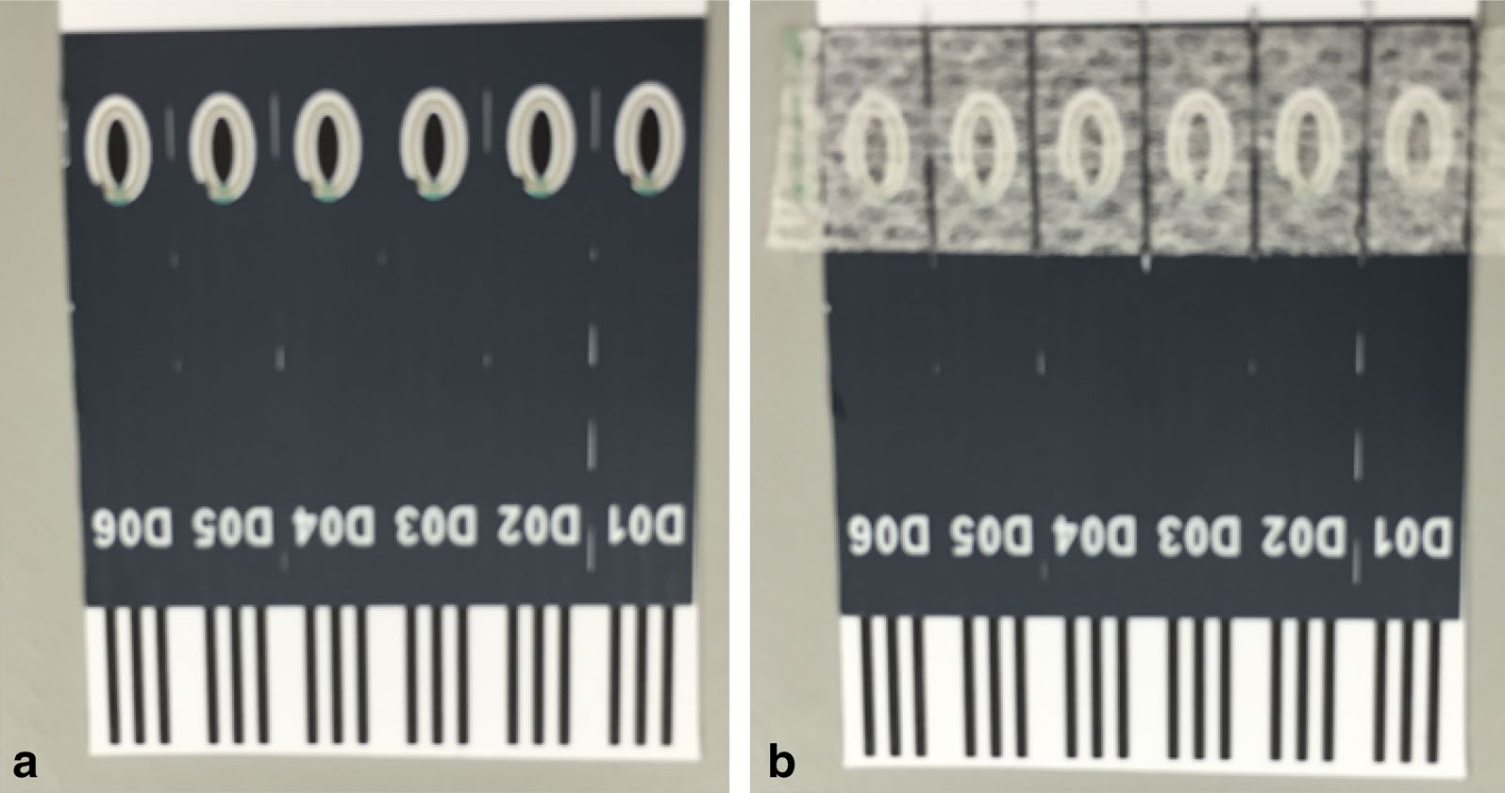
Screen printed electrode (**a**) without and (**b**) with overlayer applied. The sensor comprised the ovalular carbon working electrode (3.2 mm length, 1.2 mm width) and outer concentric Ag/AgCl counter/reference electrode

The cyclic voltammetry of OX0245 is shown in Fig. 3, using screen printed sensors with the format shown in Fig. 4a. The sensors consisted of a two electrode system using a carbon working electrode surrounded by a combined Ag/AgCl reference/counter electrode. The parent mediator shows good reversible electrochemistry, undergoing oxidation/reduction at +0.059 V and −0.005 V. The reduction peak height increased by 25 % in the presence of 100 ug/mL Δ^9^-THC.

### Sensor construct

For a simple disposable device it was desired that the mediator and buffer solution be dried down in some way on the sensor. One way of achieving this would be to deposit mediator solution directly onto the sensor and evaporate the solvent, so that on application of sample the mediator would dissolve into the sample. Alternatively, the mediator solution could be dried onto a porous overlayer which is then secured over the sensor. On application of sample, the mediator dissolves and diffuses to the working electrode where it can undergo reaction. Deposition requires tight control of the volume and position of the dispensed reagent, therefore an overlayer is preferred and was used in the prototype sensor.

The sensor was constructed by placing a dried reagent overlayer containing mediator, buffer, salt and surfactant over the electrodes, as shown in Fig. 4b. On applying sample to one end of the membrane, the sample wicked along the overlayer, wetting the reagents and the electrode surfaces. OX0245 has good solubility of at least 1 mg/mL in pH 9.5 buffer, and hence it dissolved off the overlayer rapidly.

### Initial electrochemical procedure

Initially the procedure consisted of (1) trigger; (2) wait time; (3) galvanostatic oxidation (G) and (4) chronoamperometric reduction (CA).

The test procedure was commenced with an electrochemical trigger, using the cut-off function within the Nova software of the Autolab instrument. The cut-off consisted of an applied potential of −0.3 V with a time limit of 100 s. The next step of the procedure was triggered when the working electrode current was greater than −100 nA, which typically took 5–10 s after application of sample to the overlayer.

The sensor was used in a two electrode format with a combined counter/reference electrode since it was found that the sensor was more uniformally wetted at the trigger time with a two electrode system compared to a three electrode system, where the sensor was sometimes only partially wetted.

The trigger was followed by a wait time, to allow the dried reagents on the overlayer to fully wet up and reach the electrode. During the wait time, the working electrode was at open circuit potential, which typically stabilized at –0.04 V. It was found that more viscous saliva samples took longer to completely wet the overlayer, and a wait time of 20 s was chosen to ensure complete wetting of the overlayer.

The wait time was followed by G for 5 s and CA at −0.2 V for 2 s. Galvanostatic oxidation was selected in preference to potentiostatic oxidation since the mediator concentration adjacent to the working electrode may vary from sensor to sensor, since it will be dependent on the consistency of coverage of mediator on the overlayer and the rate of diffusion of reduced mediator from the overlayer to the electrode surface, which in turn can vary with the viscosity of the saliva sample. Variation in the mediator concentration at the electrode surface will result in a variable amount of oxidized mediator being produced during potentiostatic oxidation, since the rate of oxidation will be dependent directly on mediator concentration as described by the Cottrell equation.

Galvanostatic oxidation requires there to be sufficient mediator present to ensure oxidation of only the mediator. Insufficient mediator would result in oxidation of any other oxidizable species present, such as phenolic groups at the carbon electrode surface. Therefore a high mediator loading of 1 mg/mL was used on the overlayer. The magnitude of the shift in potential of the working electrode during the G step gives an indication of whether there is sufficient mediator available. Excessively large potential shifts indicate insufficient mediator.

There are relatively few examples of galvanostatic oxidation to generate reactant in the literature. Tomcik et al. have reported the galvanostatic generation of hypobromite at an interdigitated microelectrode array, for end-point titration of the drugs Antabus and Celaskon, although this used separate generator-collector electrodes [16]. In our application, the working electrode is used to both generate the reactant (oxidized mediator) and detect the mediator -THC adduct.

**Fig. 5:**
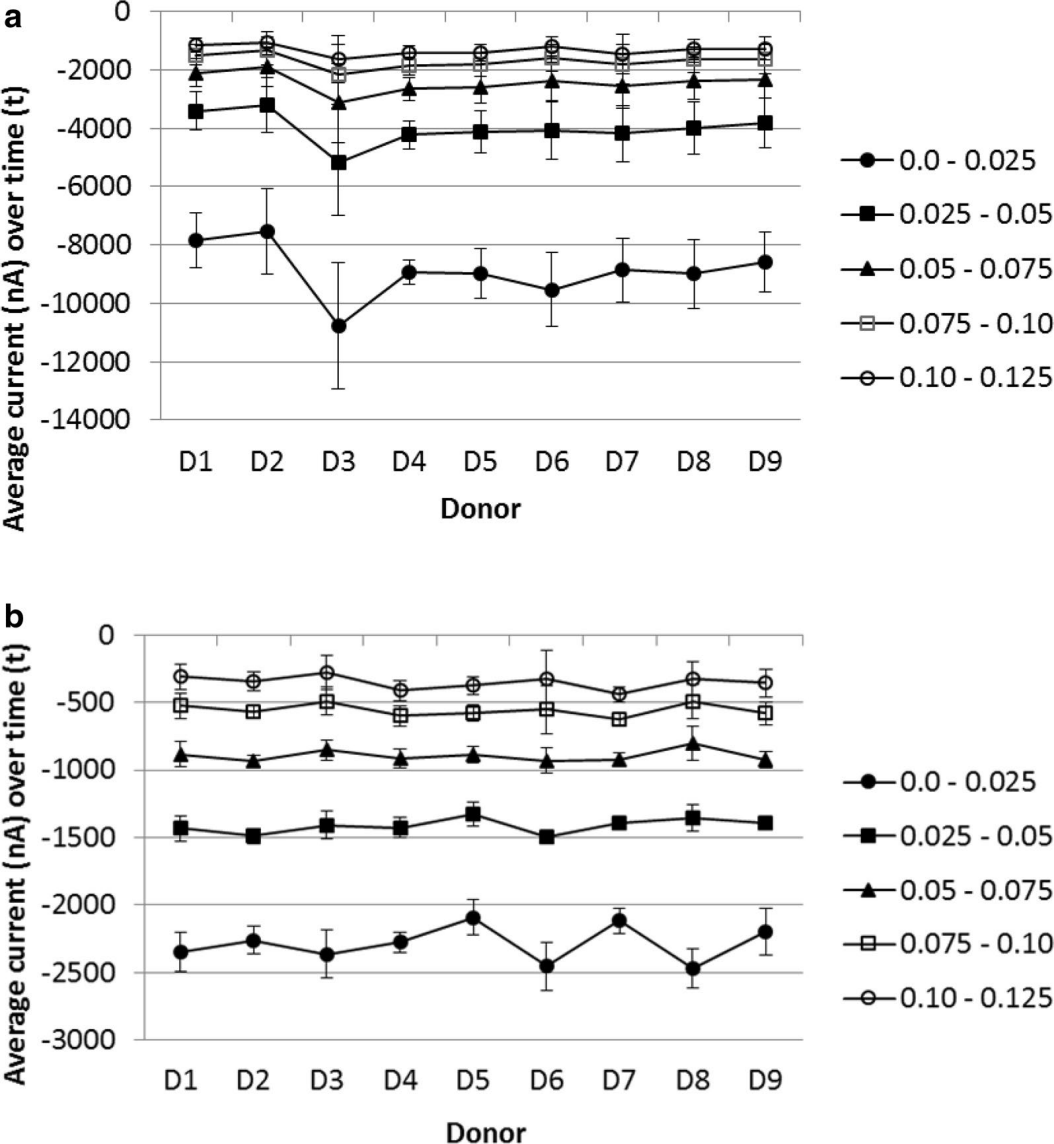
Response to saliva from nine donors using G-CA or CA1-CA2-G-CA3. **a** CA response from G-CA and **b** CA3-CA1 response from CA1-CA2-G-CA3. Each data point is the average response of 12 sensors, averaged over the time intervals 0.0–0.025, 0.025−0.05, 0.05−0.075, 0.075−0.1 and 0.1–0.125 s. The* error bars* are one standard deviation. The overlayer was coated with 1 mg/mL OX0245 in 0.4 M AMPSO (pH 9.5), 1 M NaCl, 1 % TX-100 and 0.5 % Surfynol 465. 7 uL of saliva obtained from one donor was applied to each sensor. The G-CA procedure was 20 s wait time, galvanostatic current of 100 nA for 5 s, followed by chronoamperometric reduction at −0.2 V for 2 s. The CA1-CA2-G-CA3 procedure was 20 s wait time, CA1 at −0.2 V for 2 s, CA2 at −0.04 V for 0.5 s, galvanostatic current of 100 nA for 5 s, followed by CA3 at −0.2 V for 2 s

The response to saliva obtained from nine donors using G-CA is shown in Fig. 5a. The procedure used (1) trigger; (2) wait time; (3) G of 100 nA for 5 s and (4) CA at −0.2 V for 2 s. For each sensor response, the average CA current during the specified time periods was calculated. Each sample was tested with several sensors, and the average of these sensor responses over the specified time periods is shown in the Figure. There is some variation in the chronoamperometric responses observed between samples from different donors. This was thought due to interferents in the samples producing an extra reduction current, and possibly also due to variations in the sensor construct.

### Optimization of electrochemical procedure

To overcome the donor variation in response, an extra initial chronoamperometric step was introduced, at the same potential as the final chronoamperometric step, so that subtraction of the first transient current response from the final transient current response would correct for any interferent response. However this first chronoamperometric step also introduced some variability in final CA response, since the first CA step took place at −0.2 V, whereas the open circuit potential at the start of the galvanostatic step was typically −0.04 V. It was found that on switching to open circuit potential from an applied potential of −0.2 V, it took approximately 2 s for the electrode potential during the galvanostatic step to reach −0.04 V. It is unknown whether any species were being oxidized during this period, and it may be that oxidation was primarily of surface groups on the carbon electrode.

Consequently an extra CA step was introduced between the first CA step and the G step. This second chronoamperometric step was at −0.04 V and of 0.5 s duration, the intention being to poise the electrode potential at the typical open circuit potential for the start of the galvanostatic step.

The final electrochemical procedure was as follows: (1) trigger; (2) wait time of 20 s; (3) CA1 at −0.2 V for 2 s; (4) CA2 at −0.04 V for 0.5 s; (5) G at 100 nA for 5 s and (6) CA3 at −0.2 V for 2 s. Subtraction of CA1 from CA3 for each sensor should allow correction for any interferent response.

The response to saliva from nine donors using CA1-CA2-G-CA3 is shown in Fig. 5b (these are the same saliva samples used in Fig. 5a). There was a significant reduction in %CV when using CA3-CA1 currents obtained using CA1-CA2-G-CA3, compared to the CA current using CA-G, as shown in Table 1.

**Table 1:** %CV of response, all donors, for the responses in Fig. 5

	%CV all donor responses, using average current over time (t)				
	0.0–0.025	0.025–0.05	0.05–0.075	0.075–0.10	0.10–0.125
A	−16.3	−26.5	−30.9	−32.8	−34.3
B	−7.9	−6.2	−10.2	−18.6	−32.3

**Fig. 6:**
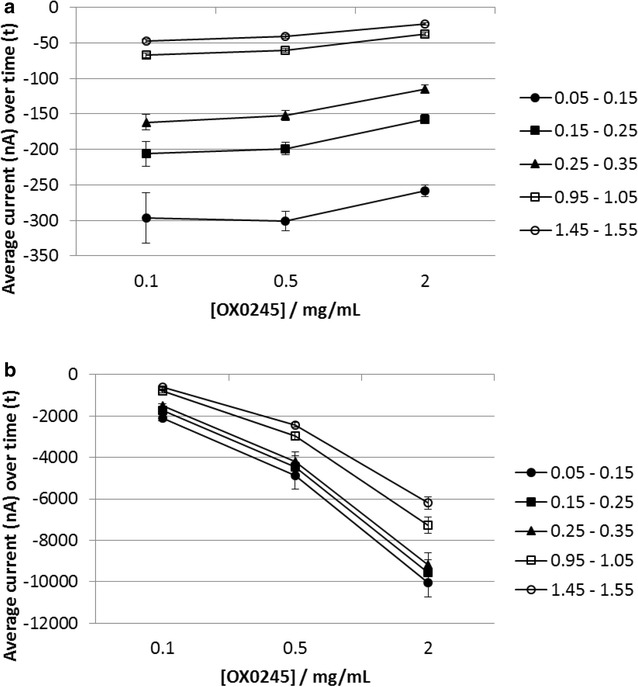
Chronoamperometric reduction response to OX0245, using potentiostatic or galvanostatic oxidation. Each data point represents the average current response obtained from 6 sensors, averaged over the time intervals 0.05–0.15, 0.15–0.25, 0.25–0.35, 0.95–1.05 and 1.45–1.55 s. *Error bars* are one standard deviation. 15 uL of solution containing 0.1, 0.5 or 2 mg/mL OX0245 in 0.04 M AMPSO solution (pH 9.5) 0.1 M NaCl and 0.002 % TX-100 was pipetted onto a sensor. The procedure used a 5 s wait time, then **a** galvanostatic oxidation at 100 nA for 5 s or **b** potentiostatic oxidation at +0.3 V for 5 s, followed by chronoamperometric reduction at −0.1 V for 2 s, with a sample rate of 2.5 ms

The advantage of G is demonstrated in Fig. 6, which shows the CA3-CA1 response to different concentrations of OX0245 when using potentiostatic or galvanostatic oxidation. On increasing the mediator concentration from 0.1 to 2 mg/mL there is a 380 % increase in chronoamperometric (CA) current at the first time point (0.05–0.15 s) when using potentiostatic oxidation, compared to a 13 % decrease in CA current when using galvanostatic oxidation.

**Fig. 7:**
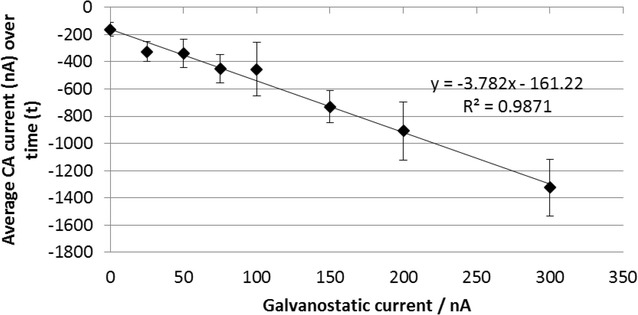
Effect of varying the magnitude of the galvanostatic current on the CA3-CA1 response. Each data point is the average current response of 6 sensors averaged over 0.05–0.15 s. The* error bars* are one standard deviation. The overlayer was treated with 1 mg/mL OX0245 in 0.4 M AMPSO (pH 9.5), 1 M NaCl, 1 % TX-100 and 0.5 % Surfynol 465. 7 uL of saliva obtained from one donor was applied to each sensor. The electrochemical protocol was as follows: 5 s wait time, CA1 at −0.2 V for 2 s, CA2 at −0.04 V for 0.5 s, galvanostatic current of 0–300 nA for 5 s, followed by CA3 at −0.2 V for 2 s

The effect of the magnitude of the galvanostatic current on CA3-CA1 response was investigated using saliva from a single donor, shown in Fig. 7. The CA3-CA1 current was linearly dependent on the galvanostatic current from 25 to 300 nA, showing that good control of the oxidation process was occurring. A galvanostatic current of 100 nA was selected since this gave a measurable CA response compared to no G, while a higher galvanostatic current would result in higher baseline CA current against which the response to Δ^9^-THC would have to be determined. This could reduce the accuracy of the device if the Δ^9^-THC response was a small current change on a large background current response obtained in the absence of Δ^9^-THC.

### Response to Δ^9^-THC in undiluted saliva

**Fig. 8:**
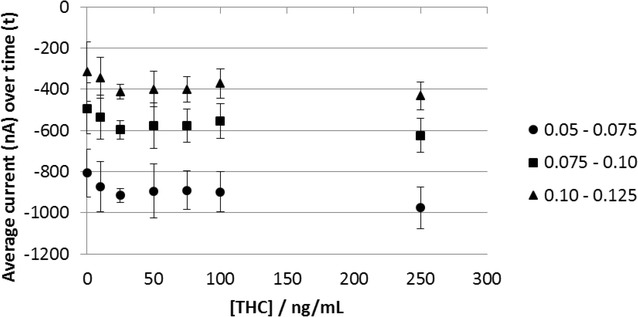
Chronoamperometric response to saliva spiked with Δ^9^-THC. Each data point is the average response of 12 sensors averaged over 0.05–0.075, 0.075–0.1 and 0.1–0.125 s. The* error bars* are one standard deviation. The overlayer was treated with 1 mg/mL OX0245 in 0.4 M AMPSO (pH 9.5), 1 M NaCl, 1 % TX-100 and 0.5 % Surfynol 465. 7 uL of sample was applied to the overlayer. The electrochemical protocol was as follows: 20 s wait time, CA1 at −0.2 V for 2 s, CA2 at −0.04 V for 0.5 s, galvanostatic current of 100 nA for 5 s, followed by CA3 at −0.2 V for 2 s

The CA3-CA1 response to saliva from a single donor spiked with Δ^9^-THC in 10 % methanol using the CA1-CA2-G-CA3 procedure is shown in Fig. 8. There is a small increase in current in response to 10 and 25 ng/mL THC compared to 0 ng/mL. The current response then shows no change between 25 and 100 ng/mL, although there is possibly a further small decrease at 250 ng/mL.

It would appear that the sensor is sensitive to low concentrations of Δ^9^-THC. This may be a reflection of the availability of Δ^9^-THC for reaction with the oxidized mediator. Δ^9^-THC is lipophilic and is largely protein bound in biological fluids. Although the overlayer contains Triton and Surfynol surfactants, these may be insufficient or too slow acting to release all the Δ^9^-THC from protein within the response time of the sensor.

### Trial of the sensors using fresh samples from cannabis smoking donors

**Fig. 9:**
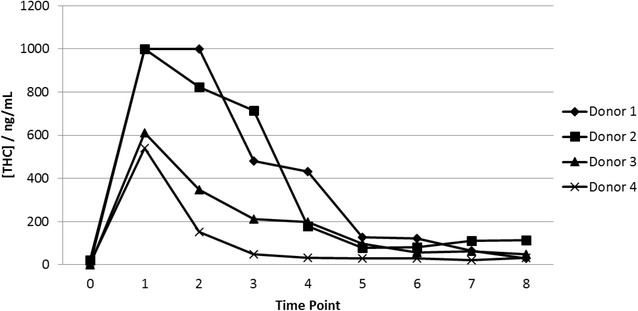
Saliva Δ^9^-THC concentrations determined by LC/MSMS for samples from the clinical trial. The samples were obtained from four cannabis smoking donors. Time point 0 was before smoking cannabis and the donors smoked a cannabis cigarette between time points 0 and 1. Time points 1–8 were at 30 min intervals. The upper detection limit of the assay was 1000 ng/mL

A trial of the sensors was conducted at SWOV Institute for Road Safety Research in The Hague using saliva collected from four cannabis smoking volunteers. The Δ^9^-THC concentrations determined by LC/MSMS of the samples collected from the cannabis smoking donors are shown in Fig. 9. The samples show a typical time dependent response after smoking. The samples contained a range of Δ^9^-THC concentrations with which the sensors could be tested. Fourteen samples were also collected from non-smoking donors which had negligible concentrations of Δ^9^-THC.

**Fig. 10:**
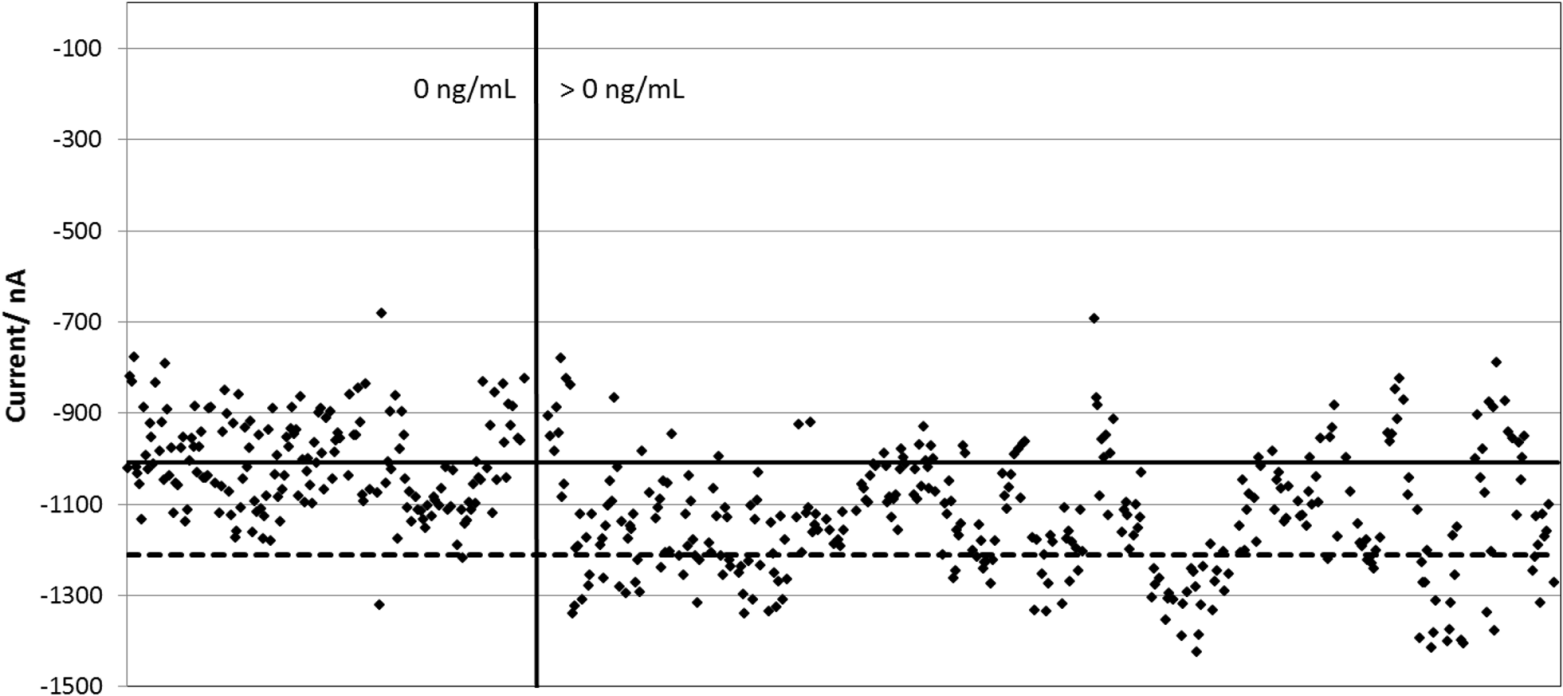
Clinical trial results obtained from 4 cannabis smoking donors and 16 non-smoking donors. Each data point is the CA3-CA1 current response for one sensor, using the average chronoamperometric transient current response between 0.05–0.075 s. Each sample was tested with 12 sensors. The solid horizontal line is the average current value of the samples with 0 ng/mL THC. The dotted horizontal line is two standard deviations from the average current of the 0 ng/mL samples. The sensor format and electrochemical sequence were as described in Fig. 8

Each sample was designated negative (0 ng/mL) or positive (>0 ng/mL) according to the LC/MSMS results. The sensor performance was characterized for sensitivity, specificity and accuracy of response.

A cut-off value for the current was calculated using the CA3-CA1 current responses for samples containing 0 ng/mL Δ^9^-THC. The cut-off was defined as the average CA3-CA1 current plus 2 standard deviations. Based on this cut-off and the sample Δ^9^-THC concentrations determined by LC/MSMS, each individual sensor response was assigned as either TN, TP, FN or FP (true negative, true positive, false negative or false positive) i.e. a true negative or false positive sensor response had a sample Δ^9^-THC concentration of 0 ng/mL as determined by LC/MSMS and current response either below or above the cut-off; a true positive or false negative sensor response had a sample Δ^9^-THC concentration of >0 ng/mL as determined by LC/MSMS and current response above or below the cut-off (Fig. 10).

The device sensitivity, selectivity and accuracy were defined as:

- Sensitivity = 100 × TN/(TN + FP)
- Selectivity = 100 × TP/(TP + FN)
- Accuracy = 100 × (TN + TP)/(TN + TP + FN + FP)

Table 2 shows the sensor performance at different time points on the CA3-CA1 response.

**Table 2:** Sensor performance at different time points on the CA3-CA1 response

Time/s	0.000–0.025	0.025–0.050	0.050–0.075	0.075–0.100	0.100–0.125	0.125–0.150	0.150–0.175	0.175–0.200	0.200–0.225	0.225–0.250
False positives	1	2	2	1	1	1	1	1	1	0
True negatives	172	168	169	168	166	165	165	167	165	165
True positives	14	95	95	68	39	30	22	20	15	14
False negatives	328	248	242	264	299	300	312	313	313	309
% Sensitivity	4	28	28	20	12	9	7	6	5	4
% Specificity	99	99	99	99	99	99	99	99	99	100
% Accuracy	36	51	52	47	41	39	37	37	36	37

Note that outlier responses were removed from the analysis. Outliers were defined as (*1*) did not trigger; (*2*) excessively noisy response resulting in atypical transient shape and (*3*) statistical outlier for each set of 12 sensors tested per sample, defined as outside 1.5x the interquartile range from the median current response

It can be seen from Table 2 there was a sweet spot for maximum sensitivity of response to Δ^9^-THC at 0.05–0.075 s giving sensitivity, selectivity and accuracy of 28, 99 and 52 % respectively. The number of false positives was very low i.e. samples containing no Δ^9^-THC were accurately assigned. However the number of false negatives was high, reflecting the relatively small concentration range within which the sensor responds to Δ^9^-THC.

### Experimental

Δ^9^-THC was purchased as 1 mg/mL solution in methanol (Cerilliant, T-005) and the mediator OX0245 (PH010250) were obtained from Sigma-Aldrich Co. Ltd (Poole, UK). All other chemicals were purchased from Sigma-Aldrich Co. Ltd. All chemicals were used as received without further purification. All solutions were prepared using deionized water with resistivity no less than 18.2 MΩ cm.

Screen printed electrodes were fabricated in house with appropriate stencil designs using a DEK horizon printing machine (DEK, Weymouth, UK). Successive layers of carbon-graphite ink (C2110406D4), dielectric ink (D2070423P5) and Ag/AgCl ink (60:40, C2030812P3) obtained from Gwent Electronic Materials Ltd. (Pontypool, UK) were printed onto a polyester substrate. The layers were cured using a tunnel drier at 70 °C (Natgraph, Nottingham, UK).

The overlayer material was composed of abaca and cellulosic fibres (75 %) in a polypropylene thermoplastic matrix (25 %), dry weight 16.5 g/m^2^ (CD020010, Ahlstrom) in reel format (1 cm wide) was obtained from Ahlstrom (Duns, UK). The overlayer was coated with OX0245 as follows: 1 mg/mL OXO245 was prepared in 0.4 M AMPSO buffer solution (pH 9.5) containing 1 M NaCl, 1 % Triton X-100 and 0.5 % Surfynol 465. The solution was dispensed onto the membrane at a loading of 0.1–1 mg/mL and dried at 40 °C. The dried overlayer was heat soldered to each sensor along the edges.

Voltammetric measurements were performed using a multiautolab M101 (Eco Chemie) potentiostat. The screen printed sensors were used as a two electrode system, with a combined counter/reference electrode (Ag/AgCl ink). The sensor format is shown in Fig. 4 with and without the overlayer.

Each saliva sample was collected immediately before use by spitting into a pot. Saliva sample containing Δ^9^-THC was prepared by firstly dispensing a known volume of 10 ug/mL Δ^9^-THC/methanol into a glass vial evaporating the methanol, then adding a known volume of saliva to achieve the required final THC concentration. The glass vial was then placed on a roller mixer for at least 1 h to dissolve the Δ^9^-THC before use. A 1 mL aliquot of each Δ^9^-THC/saliva sample was pipetted into a Quantisal saliva collection device (Agriyork 400 Ltd, Pocklington, UK) and sent for quantitative analysis by LC/MSMS by Synergy Health (Gwent, UK). The assay reportable range was <0.25–1000 ng/mL.

Saliva buffer, which mimics real saliva except for the absence of proteins, consisted of 27.5 mM sodium chloride, 6.3 mM ammonium chloride, 4.9 mM sodium phosphate (monobasic), 2.9 mM potassium chloride, 1.1 mM sodium citrate (anhydrous), 0.02 mM magnesium chloride (anhydrous), 0.27 mM sodium carbonate and 0.2 mM calcium chloride. Artificial saliva was prepared using saliva buffer with the addition of 0.3 mg/mL human recombinant lysozyme (Sigma, L1667) and 0.021 mg/mL mucin from bovine submaxillary glands (Sigma, M3895).

A trial of the sensors was conducted with fresh samples from cannabis smokers at SWOV Institute for Road Safety Research, The Hague, Netherlands. Ethical consent was obtained for the trial. Prior to smoking, a saliva sample was obtained from each of four donors. Each donor then smoked a cannabis cigarette containing an unknown quantity of THC. After smoking saliva samples were collected from each donor at 30 min intervals for the following 4 h. The donors were allowed to sip water during the trial. Sixteen samples were also collected from non-smoking donors. A 1 mL aliquot of each sample was placed in a Quantisal collection tube and sent for analysis of Δ^9^-THC content using LC/MSMS by Synergy Health.

## Conclusions

The detection of 25–50 ng/mL Δ^9^-THC in undiluted saliva has been reported using mediated disposable screen printed sensors with a response time of 30 s. The sensors used a triple chronoamperometric method combined with galvanostatic oxidation of the mediator to reduce the effect of donor variation in response, however some variation remained and further optimization of the sensor is required.

## Abbreviations

Δ^9^-THC: Δ^9^-Tetrahydrocannabinol
OX0245: N-(4-amino-3-methoxyphenyl)-methanesulfonamide
CA: chronoamperometric reduction
G: galvanostatic oxidation
% CV: % coefficient of variation
TN: true negative
TP: true positive
FN: false negative
FP: false positive
